# The views of psychiatrists on proposed changes to the England and Wales Mental Health Act 1983 legislation for people with intellectual disability: A national study

**DOI:** 10.1177/00207640231212112

**Published:** 2023-11-24

**Authors:** Samuel Tromans, Gemma Robinson, Alexandra Gabrielsson, Paul Bassett, Indermeet Sawhney, Paraskevi Triantafyllopoulou, Angela Hassiotis, Rohit Shankar

**Affiliations:** 1 Department of Population Health Sciences, University of Leicester, UK; 2 Adult Learning Disability Service, Leicestershire Partnership NHS Trust, UK; 3 Forensic Community Learning Disability Team, Southern Health NHS Foundation Trust, Calmore, UK; 4 Hertfordshire Partnership University NHS Trust, Hatfield, UK; 5 Statsconsultancy Ltd, Bucks, UK; 6 Tizard Centre, University of Kent, Canterbury, UK; 7 University College London, UK; 8 University of Plymouth Peninsula School of Medicine, UK; 9 Cornwall Partnership NHS Foundation Trust, Truro, UK

**Keywords:** Mental Health Act, developmental disability, behaviours that challenge, mental disorders, detention, sectioning

## Abstract

**Background:**

The Draft Mental Health Bill proposes removal of both intellectual disability and autism from Section 3 of the Mental Health Act for England and Wales (MHA). This would lead to people with intellectual disability (PwID) and/or autism could not be detained beyond 28 days, in the absence of diagnosed co-occurring mental illness.

**Aim:**

To obtain views of psychiatrists working with PwID in England and Wales regarding the proposed MHA changes. This study focusses specifically on the impact on PwID.

**Methods:**

A cross-sectional online mixed methodology survey of Likert and free-text response questions was developed, to ascertain perceptions of proposed legislative changes to the MHA. A non-discriminatory exponential snowballing technique leading to non-probability sampling was used to disseminate the survey. Quantitative data was analysed using descriptive statistics, Mann-Whitney and Fisher’s exact tests. Thematic analysis was conducted on free text responses.

**Results:**

A total of 82 psychiatrists (33%) from approximately 250 eligible completed the survey. Nearly two-thirds (64%) reported good awareness of the proposed changes, with over half (55%) reporting disagreement with the changes. Psychiatrists working in inpatient settings for PwID reported increased awareness of the changes, less agreement with the reforms, and increased expectations of the reforms having negative unintended consequences, compared to their peers working exclusively in the community. Consultants reported greater disagreement with the changes compared to their non-consultant peers. Qualitative analysis identified five main themes: impact on diagnosis and treatment, seeking alternative options, introducing inequities, resources, and meeting holistic care goals through the Care, Education and Treatment Reviews (CETR) process.

**Conclusion:**

Psychiatrists working with PwID report widespread disagreement with the proposed changes to the MHA for PwID, with greater levels of disagreement among those working in inpatient services. Caution with respect to the proposed changes, and monitoring of the impact of the changes if implemented, is advised.

## Introduction

Compared to the general population, people with intellectual disability (PwID, also called ‘learning disability’) are at heightened risk of having a mental illness compared to their peers (Cooper et al., 2007). Furthermore, PwID have a higher likelihood of being autistic, particularly those with moderate to profound ID (Brugha et al., 2016), and autism is similarly associated with a heightened risk of mental illness (Lai et al., 2019).

### The Mental Health Act (MHA) 1983 for England and Wales and its Current Application to PwID

The MHA defines ‘mental disorder’ as ‘any disorder or disability of the mind’ (Mental Health Act, 1983). Furthermore, ‘a person with learning disability shall not be considered by reason of that disability to be suffering from mental disorder for the purposes of the provision or requiring treatment in hospital for mental disorder, unless that disability is associated with abnormally aggressive or seriously irresponsible conduct on their part’ (Mental Health Act, 1983). It is under this classification that PwID and/or autism who do not present with concurrent major mental illness, may currently be detained in hospital for assessment and treatment under Part II of the MHA, colloquially known as ‘Civil Sections’, either under Section 2 (Admission for Assessment) or Section 3 (Admission for Treatment) (Mental Health Act, 1983). Part III of the MHA covers what are colloquially known as the ‘Forensic Sections’, allowing those with a mental disorder who are within the criminal justice system to be detained, often in a secure hospital (Mental Health Act, 1983).

### Current Utility of the MHA for PwID

There has been a sharp reduction in inpatient National Health Service beds for PwID in England over recent decades (Devapriam et al., 2015), from 33,421 beds in 1987 to 1988, to 771 in 2022 to 2023, representing a 97.7% decrease (NHS England, 2023). Recent data collected by NHS England reports that between 1st and 30th April 2023 there were 2,060 PwID and/or Autism currently residing in inpatient beds in England, 1,200 of whom had a documented diagnosis of ID (NHS Digital, 2023). Of these patients, 1,890 (92%) were detained under the framework of the MHA; 1,095 under Part II (Civil Sections) and 755 under Part III (Forensic Sections). This predominance of the use of Civil Sections has been present since recordings of this dataset began in 2015. The average length of stay for these patients as of April 2023 was 990 days (NHS Digital, 2023).

### The Mental Capacity Act (2005) and Its Relevance to the MHA With Regard to PwID

The Mental Capacity Act (MCA) is the second most-commonly used legislation utilised to detain PwID in hospital (Series, 2019b), most often facilitated through the use of Deprivation of Liberty Safeguards (DoLS). These allow for those with impaired decision-making capacity, who are not objecting to their care and treatment, to be deprived of their liberty in hospital (Mental Capacity Act, 2005). The exact number of people detained in hospital under the MCA is unclear, due to the large number of unprocessed DoLS applications and the lack of consistent recordings of ID on these applications (Series, 2019a) .There is a lack of various legal safeguards such as Tribunal and Second Opinion reviews and Nearest Relative rights, which are built into the MHA (Series, 2019b). The use of the MCA to detain PwID in hospital in England has been widely criticised.

### Proposed Changes to the MHA With Regard to PwID

The Draft Bill to amend the MHA, published in June 2022 (Draft Mental Health Bill, 2022), makes proposed amendments to the Act, including the exclusion of PwID (and autism) from the grounds for detention under selected sections of the Act.

The narrative for change to the MHA and its application for PwID can be seen through numerous reports and inquiries over the past three decades which have highlighted the need for good quality commissioning and service provision for PwID (Department of Health, 2001; Mansell, 2007), the need for short-term admissions focussed on high quality assessment and treatment (NHS England, Association of Directors of Adult Social Services & Local Government Association, 2015), a focus on improving community services (Mansell, 2007; NHS England, Association of Directors of Adult Social Services & Local Government Association, 2015) and preventing the so-termed ‘warehousing’ of PwID in hospital (Department of Health, 2012). One of the proposals put forward by campaigners to prevent the warehousing of PwID has been to remove PwID and autism from Section 3 of the MHA, making them ineligible for detention under these grounds alone (National Autistic Society, 2022; Wood, 2020).

The Draft Bill (Draft Mental Health Bill, 2022), intended to modernise the MHA and improve the way PwID and/or autism are treated in law, proposes the removal of eligibility to be detained under Section 3 of the Act (Admission for Treatment) on the basis of ID and/or autism alone, although retaining the ability to detain under Section 2 and Part III of the Act. In addition, proposals have been made to provide a ‘statutory footing’ for Care and Treatment Reviews (CTRs) (Draft Mental Health Bill, 2022) and enhance the statutory duty of commissioners to ‘provide enough support’ (Draft Mental Health Bill: Explanatory Notes, 2022).

### The MHA Changes for PwID in the 1990s in New Zealand

Similar legislative changes had been made previously in New Zealand, a jurisdiction with a comparable ‘common law based’ legal system to the UK. Changes to New Zealand’s Mental Health legislation in 1992 led to the explicit exclusion of this cohort of patients from the detention powers (Mental Health (Compulsory Assessment and Treatment) Act, 1992) in an attempt to ‘encourage choice and independence’ (McCarthy & Duff, 2019).

Subsequent scrutiny of these changes highlighted a ‘legislative gap that significantly limited the options available to the courts’ and ‘closed a civil entry for PwID who might present with risk behaviours not necessarily escalating to the criminal justice system’ (McCarthy & Duff, 2019). Options left available for PwID and challenging and/or offending behaviours in New Zealand included leaving them in the community, admitting them to a forensic hospital as a ‘special patient’ or sending them to prison; a situation which required the development of additional legal frameworks in 2004 (Intellectual Disability (Compulsory Care and Rehabilitation) Act, 2003), to address the gap in services which had been created by removing this cohort’s eligibility (McCarthy & Duff, 2019).

### The MHA and PwID in Other Countries With Similar Models of Health and Legal Systems

There is considerable global variation with respect to how PwID and autistic people are treated within their MHA-equivalent legislative frameworks. In many countries, there are different versions of mental health legislation for different regions, such as in Australia (Mental Health Act, 2014) and Canada (Mental Health Act, 1996). Additionally, many nations do not specifically mention autism within their legislation, such as for India (The Mental Healthcare Act, 2017), Nigeria (National Mental Health Act, 2021), and Scotland (Mental Health (Care and Treatment) (Scotland) Act, 2003), leaving it open to interpretation as to whether autism falls within their respective definitions of mental disorder. With respect to PwID, some national and regional legislations do not consider it to fall within their definitions of mental disorder, such as for India (The Mental Healthcare Act, 2017), the Victoria region of Australia (Mental Health Act, 2014), and the province of British Columbia in Canada (British Columbia Ministry of Health, 2005), with the latter legislation explicitly stating that intellectual disabilities alone is insufficient justification for admission under their Act. However, for Ireland (Mental Health Act, 2001), Nigeria (National Mental Health Act, 2021), and Scotland (Mental Health (Care and Treatment) (Scotland) Act, 2003), intellectual disabilities fall within their respective definitions of mental disorder.

### Other Identified Concerns

As England and Wales debate changes to mental health legislation that are comparable to those previously implemented in New Zealand, similar and additional concerns have been brought to the attention of the Parliamentary Joint Committee, who have been tasked with appraising the Draft Bill (Parliamentlive.tv, 2022). These concerns have highlighted potential risks to patients in removing the eligibility to be detained under Section 3 of the Act, including the risk of misdiagnosis, misdirection into the criminal justice system and a mis-use of alternative legal frameworks (Parliamentlive.tv, 2022).

Healthcare professionals and researchers have warned of the potential human rights implications of removing a ‘suitable legal structure’ from a vulnerable cohort of patients and have highlighted that consideration of the issues with all stakeholders, including psychiatrists, is necessary to address the complexity of the issues (Courtenay, 2021).

### Aim

The aim of this study was to obtain the views to the proposed legislative changes to the MHA of psychiatrists working with PwID in England and Wales. For the purposes of this study, we focussed specifically on PwID rather than autistic people

## Methodology

The STROBE Guidance for cross sectional studies were followed (Supplemental Information 1).

### Survey Development

The survey questions were developed collaboratively by the authors, with the resulting online survey being produced on the Google Forms online platform. Survey questions related to obtaining data on the professional demographic details of participants, as well as their familiarity and views relating to the proposed changes to the MHA. The survey is available in the Supplemental Information 2. The Survey had 19 questions of which 14 were Likert style and five inviting free text opinions. The questions captured the respondent’s professional demographic details, knowledge of ongoing debate on the changes to the MHA and views on the overall and specific changes under consideration.

### Ethics and Governance

The NHS Health research authority tool (http://www.hra-decisiontools.org.uk/research/index.html) confirmed no formal NHS Ethics approval was required (supplemental Information 3). All participants were advised at the start of the study that participation was voluntary and their replies, if they chose to participate, would be anonymised and analysed. No participant identifier data was collected. Data was pooled prior to analysis. Further, it was to a professional participant group where consent was implicit by participation. It was specified that informed consent would be presumed if participants submit the survey.

### Participants and Recruitment

Survey invitations were disseminated to psychiatrists working with PwID across England and Wales through the authors networks who are closely linked to the Intellectual Disability faculty of the Royal College of Psychiatrists. It is envisaged that there are approximately 250 psychiatrists working substantively or in a training role in services for PwID in England and Wales. After the survey was open for 6 weeks two further reminders were sent out. The survey opened on 19th February 2023 and closed on 10th May 2023. On average, it was estimated that it took participants 8 to 10 min to complete the questionnaire.

### Data Analysis

#### Analysis of Quantitative Measures

SPSS 26 was used to analyse the survey data. Descriptive statistics were used to calculate frequencies of respondents’ characteristics and answer categories as per the multiple-choice questions.

Two sets of subgroup analyses were performed. The first analysis compared the responses of consultants against clinicians with non-consultant roles, including speciality and associate specialist (SAS) doctors, as well as higher psychiatry trainees working with PwID. The second analysis compared staff working in inpatient settings (including staff working in inpatient settings only, as well as staff working in a mixture of inpatient and community settings) versus staff working exclusively in the community setting.

The majority of outcomes were ordinal in nature, consisting of either Likert-style questions on a 1 to 5 scale, or 3-point no/maybe/yes scales. Due to the nature of these outcomes, between-group comparisons were made using the Mann-Whitney test. The exception to this method of analysis was for the question item on views on the unintended consequences on the Criminal Justice System, where the responses were non-ordinal in nature. For this item, Fisher’s exact test was used for between-group comparisons.

#### Analysis of Qualitative Measures

The five qualitative/free-text survey questions sought to further explore the views, expectations and concerns of the respondents relating to the proposed MHA changes through free text responses. Analysis was conducted collaboratively between one reviewer and two senior co-authors with expertise in qualitative analysis. A descriptive thematic approach was chosen to enable the views of the respondents to be presented in a way which is applicable to everyday health care practice and suitable to mixed methodology studies (Chafe, 2017; Doyle et al., 2020; Neergaard et al., 2009). From this, five main themes were generated, and these have been summarised descriptively in the results section, along with corresponding subthemes.

## Results

### Demographic Details of Study Population

The survey received a total of 82 participant responses, with their demographic details summarised in Table 1. Two thirds of participants (*n* = 55; 67%) were consultant psychiatrists. Approximately a third of participants (*n* = 29; 35%) had over 15 years of experience of working with PwID, whilst a slightly smaller proportion (*n* = 24; 29%) had five or fewer years of experience. Of the participants, just over half (*n* = 42; 52%) were working exclusively in community settings, compared to 12% (*n* = 10) working exclusively in hospital/inpatient settings, and 36% (*n* = 29) working in both hospital and community settings. For most participants (*n* = 73; 90%), over half of their workload involved PwID. Half of the participants (*n* = 41; 50%) reported seeing more than four PwID detained under Section 3 of the MHA for abnormally aggressive or seriously irresponsible conduct without a co-occurring major mental illness in a typical year.

**Table 1.** Demographic details of participants.

Variable	*n*	Category	Number (%)
What is your role?	82	Consultant	55 (67)
		SAS	7 (9)
		Others	5 (6)
		ST4-6 (Higher trainee)	15 (18)
How many years of experience do you have working with PwID?	82	⩽5	24 (29)
		6–10	18 (22)
		11–15	11 (13)
		>15	29 (35)
Which settings do you currently work in?	81	Community (outpatient)	42 (52)
		Hospital (inpatient)	10 (12)
		Both hospital and community	29 (36)
What percentage of your working week is working with PwID?	81	⩽25	6 (7)
		26–50	2 (2)
		>50	73 (90)
In a typical year, how many PwID do you see who are detained on Section 3 MHA for abnormally aggressive or seriously irresponsible conduct?	82	None	8 (10)
		1–3	33 (40)
		4–6	21 (26)
		7–10	15 (18)
		11–20	2 (2)
		>20	3 (4)
			

### Quantitative Data Analysis

Participants were asked about their views regarding the proposed reforms to the MHA, with the majority of questions on a Likert scale. A summary of the responses to the multiple-choice question items is shown in Table 2.

**Table 2.** Summary of responses to quantitative question items.

Question item	*n*	Score	Number (%)
Prior to this survey, how aware were you of the reforms for the MHA for PwID?	82	1 (not aware)	4 (5)
		2	5 (6)
		3	20 (24)
		4	22 (26)
		5 (fully aware)	31 (38)
Agreement with MHA reform proposals for PwID, specifically the withdrawal of the criteria of abnormally aggressive or seriously irresponsible conduct for warranting compulsory treatment under Section 3 MHA	82	1 (fully disagree)	18 (22)
		2	28 (34)
		3	21 (26)
		4	11 (13)
		5 (fully agree)	4 (5)
How confident are you that the 28 days duration for detention allocated under section 2 MHA is enough time to ascertain if there is an underlying mental health condition, driving the concerning behaviour/s?	82	1 (not confident)	29 (35)
		2	24 (29)
		3	7 (9)
		4	18 (22)
		5 (fully confident)	4 (5)
How strongly do you believe behaviours in PwID which are not due to an underlying mental health disorder should require detention under Section 3 MHA if they are a risk to themselves or others?	82	1 (not at all)	9 (11)
		2	12 (15)
		3	19 (23)
		4	26 (32)
		5 (very strongly)	16 (20)
How confident are you of the proposed reforms providing adequate safeguards for PwID when they do not have a co-occurring mental health condition?	82	1 (not confident)	30 (37)
		2	29 (35)
		3	19 (23)
		4	4 (5)
		5 (fully confident)	0 (0)
Do you expect unintended consequences (negative or positive) of the proposals?	82	No	1 (1)
		Maybe	17 (21)
		Yes	64 (78)
The proposals for change are under Part II and not Part III of the act. Is this agreeable?	81	No	32 (40)
		Not sure/maybe	40 (49)
		Yes	9 (11)
Could there be unintended consequences on the Criminal Justice System as a result of the proposals to reform the MHA for PwID?	81	None	3 (4)
		Negative	40 (49)
		Negative + Positive	20 (25)
		Positive	1 (1)
		Difficult to envisage	17 (21)
The White Paper proposes a statutory requirement that the Responsible Clinician considers the findings and recommendations of Care, Education and Treatment Reviews (CETRs) in the patient’s care and treatment plan. Deviations from the recommendations should be justified and explained by the RC. What are your views on this?	80	1 (fully disagree)	18 (23)
		2	16 (20)
		3	27 (34)
		4	10 (13)
		5 (fully agree)	9 (11)

Participant responses indicated a relatively good awareness of the proposed reforms, with around two-thirds of participants (*n* = 53; 64%) scoring either 4 or 5 (on a scale where 1 corresponded to ‘not aware’ and 5 to ‘fully aware’), and only 5% (*n* = 4) of participants reporting being unaware of the changes.

More than half the respondents clearly disagreed with the proposed changes to the MHA for PwID (*n* = 46; 55%) rating this question item 1 or 2 (on a scale where 1 corresponded to ‘fully disagree’ and 5 to ‘fully agree’). Only 5% (*n* = 4) of participants reported fully agreeing with the proposed MHA reforms. There was also a relative lack of confidence that the 28-day detention period will be sufficient in ascertaining whether an underlying mental health condition is driving the challenging behaviours, with almost two-thirds of participants (*n* = 53; 64%) rating this item either 1 or 2 (on a scale where 1 corresponded to ‘not confident’ and 5 to ‘fully confident’).

Many participants also reported believing that PwID presenting with behaviours which are not due to an underlying mental health disorder should require detention under Section 3 of the MHA if they present a risk to themselves or others, with 51% (*n* = 42) rating this item as 4 or 5 (on a scale where 1 corresponded to ‘not at all’ and 5 to ‘very strongly’). Furthermore, there was a widespread lack of reported confidence that the MHA reforms will have adequate safeguards, with 72% (*n* = 59) of participants rating this item as 1 or 2, and 0% (*n* = 0) of participants rating this item as 5 (on a scale where 1 corresponds to ‘not confident’ and 5 to ‘fully confident’).

Over three quarters of participants (78%) reported expecting there to be unintended consequences of the reforms (positive or negative). Only 11% (*n* = 9) reported feeling that the MHA changes being under Part II (i.e. civil sections) and not Part III (i.e. sections through the criminal justice system) were agreeable, with around half of participants (*n* = 40; 49%) reporting ‘not sure/maybe’ in response to this item.

Three quarters of respondents (*n* = 61; 75%) thought there may be unintended consequences on the Criminal Justice System because of the proposed reforms, with the remainder not sure of the consequences. For those who did think there would be consequences, many more thought the consequences would be negative rather than positive, with 49% (*n* = 40) reporting negative consequences, 25% (*n* = 20) negative and positive consequences, and 1% (*n* = 1) positive consequences.

Finally, with respect to the proposed statutory requirement for the Responsible Clinician to consider the findings and recommendations of Care, Education and Treatment Reviews (CETRs) in the patient’s care and treatment plan, as well as justifying and explaining any deviations from the recommendations, almost half of participants (*n* = 34; 43%) rated this item as 1 or 2 (on a scale where 1 corresponds to ‘fully disagree’ and 5 to ‘fully agree’).

The first set of subgroup analyses compared between job roles. Respondents were asked to indicate their job title in one of four main categories. The analyses compared the differences in responses between consultants when compared with other (non-consultant) staff groups, including ST4-6 (Higher) trainees working with PwID, Speciality and Associate Specialist (SAS) doctors working with PwID, and others. The comparisons were between 55 responses from consultants and 27 responses from non-consultant staff. Table 3 shows the findings for consultant and non-consultant staff groups, with corresponding frequencies, percentage values, and *p*-values for each question item, indicating the statistical significance of group differences for that item.

**Table 3.** Comparison of responses of consultant versus non-consultant clinicians.

Variable	Score	Consultant		Non-consultant		*p*-Value
		*n*	*n* (%)	*n*	*n* (%)	
Awareness of reforms to MHA	1 (not aware)	55	0 (0)	27	4 (15)	.02
	2		2 (4)		3 (11)	
	3		14 (25)		6 (22)	
	4		15 (27)		7 (26)	
	5 (fully aware)		24 (44)		7 (26)	
Agreement with MHA reforms	1 (fully disagree)	55	10 (18)	27	8 (30)	.53
	2		21 (38)		7 (26)	
	3		13 (24)		8 (30)	
	4		8 (15)		3 (11)	
	5 (fully agree)		3 (5)		1 (4)	
Confidence that 28 day detention is sufficient	1 (not confident)	55	19 (35)	27	10 (37)	.90
	2		17 (31)		7 (26)	
	3		5 (9)		2 (7)	
	4		12 (22)		6 (22)	
	5 (fully confident)		2 (4)		2 (7)	
Belief of detention required undersection 3 of the MHA for behaviours in PwID which are not due to an underlying mental health disorder but if they are a risk to themselves or others	1 (not at all)	55	5 (9)	27	4 (15)	.40
	2		11 (20)		1 (4)	
	3		13 (24)		6 (22)	
	4		16 (29)		10 (37)	
	5 (very strongly)		10 (18)		6 (22)	
Confidence of reforms providing adequate safeguards	1 (not confident)	55	20 (36)	27	10 (37)	.51
	2		22 (40)		7 (26)	
	3		11 (20)		8 (30)	
	4		2 (4)		2 (7)	
	5 (fully confident)		0 (0)		0 (0)	
Unintended consequences of reforms expected	No	55	0 (1)	27	1 (4)	.02
	Maybe		8 (15)		9 (33)	
	Yes		47 (85)		17 (63)	
MHA changes agreeable	No	55	23 (42)	26	9 (35)	.79
	Not sure/maybe		25 (45)		15 (58)	
	Yes		7 (13)		2 (8)	
Unintended consequences on Criminal Justice System	None	55	3 (5)	26	0 (0)	.47
	Negative		27 (49)		13 (50)	
	Negative + Pos.		15 (27)		5 (19)	
	Positive		1 (2)		0 (0)	
	Hard to envisage		9 (16)		9 (31)	
Agree with change in White Paper	1 (fully disagree)	54	15 (28)	26	3 (12)	.003
	2		13 (24)		3 (12)	
	3		18 (33)		9 (35)	
	4		6 (11)		4 (15)	
	5 (fully agree)		2 (4)		7 (27)	

The analyses suggested statistically significant differences between the two job groups for awareness of reforms to the MHA (*p* = .02), unintended consequences of reforms (*p* = .02), and agreement with the changes in the White Paper (*p* = .003). The other questions did not significantly vary between the two groups (*p* > .05).

Consultants reported being more aware of the reforms than non-consultants. Approximately 71% of consultants (*n* = 39) responded in one of the top two awareness categories (4 or 5, on a scale of where 1 corresponds to ‘not aware’ and 5 to ‘fully aware’), compared to 52% of the non-consultant group (*n* = 28). Consultants were more likely to think that there would be unintended consequences of the reforms, with 85% (*n* = 47) reporting that there would be unintended consequences, compared to less than two-thirds (63%; *n* = 17) of the non-consultant group. Overall, consultants were significantly less likely to agree with the changes in the White Paper than non-consultants (*p* = .003). Only 15% of consultants (*n* = 8) responded in one of the top two categories (4 or 5, on a scale of where 1 corresponds to ‘fully disagree’ and 5 to ‘fully agree’), compared to 42% of non-consultants (*n* = 11).

A second set of subgroup analyses compared survey responses between those working in inpatient settings (the inpatient contact group) versus those working exclusively in community settings (the community group). The inpatient contact group consisted of participants working exclusively in hospitals, as well as those working in a mixture of hospital and community settings, whereas the community group consisted of participants working exclusively in the community. The inpatient contact group was made up of 39 participants, whilst 42 staff worked in the community only. One respondent did not indicate their work setting and was excluded from these analyses. Comparisons between the two groups were made, with the analysis results summarised in Table 4.

**Table 4.** Comparison of participant responses of clinicians with inpatient contact versus those practicing exclusively in community settings.

Variable	Score	Inpatient contact		Community		*p*-Value
		*n*	*n* (%)	*N*	*n* (%)	
Awareness of reforms to MHA	1 (not aware)	39	0 (0)	42	4 (10)	.007
	2		2 (5)		3 (7)	
	3		7 (18)		13 (31)	
	4		10 (26)		11 (26)	
	5 (fully aware)		20 (51)		11 (26)	
Agreement with MHA reforms	1 (fully disagree)	39	10 (26)	42	7 (17)	.04
	2		18 (46)		10 (24)	
	3		5 (13)		16 (38)	
	4		4 (10)		7 (17)	
	5 (fully agree)		2 (5)		2 (5)	
Confidence that 28 day detention is sufficient	1 (not confident)	39	16 (41)	42	12 (29)	.03
	2		15 (38)		9 (21)	
	3		5 (5)		5 (12)	
	4		4 (10)		14 (33)	
	5 (fully confident)		2 (5)		2 (5)	
Belief of detention required undersection 3 of the MHA for behaviours in PwID which are not due to an underlying mental health disorder but if they are a risk to themselves or others	1 (not at all)	39	2 (5)	42	7 (17)	.004
	2		6 (15)		6 (14)	
	3		4 (10)		15 (36)	
	4		16 (41)		10 (23)	
	5 (very strongly)		11 (28)		4 (10)	
Confidence of reforms providing adequate safeguards	1 (not confident)	39	19 (49)	42	10 (24)	.004
	2		14 (36)		15 (36)	
	3		6 (16)		13 (31)	
	4		0 (0)		4 (10)	
	5 (fully confident)		0 (0)		0 (0)	
Unintended consequences of reforms expected	No	39	0 (0)	42	1 (2)	<.001
	Maybe		2 (5)		15 (36)	
	Yes		37 (95)		26 (62)	
MHA changes agreeable	No	39	20 (51)	41	11 (27)	.03
	Not sure / maybe		16 (41)		24 (59)	
	Yes		3 (8)		6 (15)	
Unintended consequences on Criminal Justice System	None	38	0 (5)	42	3 (7)	.05
	Negative		24 (63)		15 (36)	
	Negative + Pos.		9 (24)		11 (26)	
	Positive		0 (0)		1 (2)	
	Hard to envisage		5 (13)		12 (29)	
Agree with change in White Paper	1 (fully disagree)	38	8 (21)	41	9 (22)	.35
	2		9 (24)		7 (17)	
	3		15 (39)		12 (29)	
	4		3 (8)		7 (17)	
	5 (fully agree)		3 (8)		6 (15)	

The results suggested statistically significant differences between those with and without inpatient contact for the majority of the questions analysed (*p* ⩽ .05). There was only slight evidence of a difference for the unintended consequences on the Criminal Justice System, where the result was of borderline statistical significance (*p* = .05). The only question that did not vary between the two groups was relating to agreement with the changes in the White Paper (*p* = .35).

Those with inpatient contact reported being more aware of the reforms (*p* = .007), with over three-quarters (77%; *n* = 30) responding in the two top agreement categories (4 or 5, on a scale where 1 corresponds 1 ‘not aware’ and 5 to ‘fully aware’), compared to only 52% (*n* = 22) of those in the community group.

For the majority of the other questions, where significant differences were observed, the group with inpatient contact were generally less in favour and more sceptical of the reforms than the community group.

Of the inpatient contact group, 72% (*n* = 28) answered in the two lowest categories on agreement with reforms (1 or 2, on a scale where 1 corresponds to ‘fully disagree’ and 5 to ‘fully agree’), compared to 40% (*n* = 17) of those in the community group (*p* = .04). Almost half of the inpatient contact group (*n* = 19; 49%) were not at all confident that reforms provided adequate safeguards (1, on a scale of where 1 corresponds to ‘not confident’ and 5 to ‘fully confident’) compared to under a quarter (*n* = 10; 24%) of the community group (*p* = .004).

Almost all (*n* = 37; 95%) of the inpatient contact group thought there would be unintended consequences of the reforms, which contrasted with 62% (*n* = 26) of the community group (*p* < .001). Over half of the inpatient contact group did not find the MHA changes agreeable (*n* = 20; 51%), compared to 27% (*n* = 11) of the community group (*p* = .03).

### Qualitative Data Analysis

Based on the survey free text responses, five overarching themes were identified, and these are described in turn below. Table 5 illustrates the themes with example comments from participants; all participant comments (with the corresponding numerical codes referred to in this section) are shown in Supplemental Information 4.

**Table 5.** Thematic analysis data table with example participant comments. Please note that the quoted text is copied verbatim from the survey, with no grammatical/spelling errors corrected.

Themes	Sample comments
1. Impact on diagnosis and treatment	
1.1. Distortion of the diagnostic process	● Section 12 approved doctors may feel inclined to exaggerate traits to the extent that a secondary mental health disorder can be proposed in order to keep the person safe in hospital.
1.2. Understanding complex presentations – need for time	● I have seen a number of cases where it has taken significantly longer than 28 days to get an understand of the causes of the aggression, understand their needs and settle them sufficiently (sometimes this has been in longer term segregation)’.
1.3. Containing risk	● As a result of inability to get the patient detained, placements may seek to involve police instead which could result in placement in custody. As a result of being held in an inappropriate environment there could be significant risks’.
2. Seeking alternative options	
2.1. Lack of safeguards	● If the MCA starts to be used more to enable PwID to access hospital treatment without the MHA option, then PwID will have fewer rights as they have no right to a Tribunal or other MHA safeguards to their admission.
2.2. Criminalisation of behaviour	● No internal logic; paradoxical diversion of vulnerable people to the prison system.
2.2.1. Accountability	● It might be entirely appropriate for PWID to get charged for their behaviour and this often doesn’t happen as they will end up in the hospital system.
3. Introducing inequities	
3.1. Discrimination and stigma	● It is discriminatory and will stigmatise people in to two categories of ‘offender’ and ‘non-offender’. Services will not become involved with a person until they have committed and offence leading to later interventions.
3.2. Impact on existing health inequalities	● People with LD will get an inferior service compared to general population’.
3.3. Equality and rights	● I think it is right that adults with learning disability should have the same rights and responsibility as other members of society so some of this reform is in the right direction if paired with appropriate support within LA, police and judicial system’.
4. Resources	
4.1. Staffing availability and expertise	● More people - staff in services. We cannot recruit to vacant posts locally for professional staff. Services need to concentrate on recruiting to current levels of provision before even contemplating expansion of services.
4.2. Community service provisions	● The main requirement is significant investment in community provisions that enable patients to be kept safely in the community. This is the common barrier to discharge and often the reason for admission in the first place.
4.3. Systems perspective	● Better links with forensic teams and ability to consult with them.
4.4. The role of social care provisions	● The biggest resource required is improved availability of skilled community placements where PwID and behaviours that challenge can be safely managed.
5. Meeting holistic care goals through the CETR process	
5.1. Disconnect between panel members and clinicians	● Extremely variable quality of panel members; fundamental unfairness in inability to challenge panel decisions; no appeals process.
5.2. Imposing recommendations	● Clinical autonomy is key for good psychiatric practice and CETR is far too prescriptive.
5.3. Shared responsibility and accountability	● The RC should explain why deviations from care plans are made, but the government need to accept that in many cases, the reason why the patient cannot be discharged is due to lack of suitable community provisions. The RC can and should only be held responsible for those elements of the care plan that he or she is empowered to be able to deal with. This would not include the sourcing and funding of community provisions’.

#### Theme 1: Impact on Diagnosis and Treatment

Respondents reported that the proposed changes to the MHA would lead psychiatrists to seeking alternative ways of negotiating admissions within the new system. This could happen through facilitating a diagnosis of mental disorder in more unconventional ways (1.1.1.) and raised concerns around over-and misdiagnosis of mental illness in PwID (1.1.2.) in order to secure and prolong stay in an inpatient unit (1.1.3). Participants emphasised that achieving insight into the patient’s presentation, obtaining a good understanding of behaviours that challenge, and confirming the presence or absence of mental disorder is often a lengthy process, requiring a longitudinal approach in the inpatient setting (1.2.3.). If such assessments were to become hurried or rushed, risks would remain high due to poorly understood or unmet needs, which inevitably would need to be managed in the community setting in the absence of an inpatient alternative (1.3.3.). Respondents envisaged that this would put high pressure onto all community provisions including police and Emergency Departments. Stakes would become high (1.3.2.) and escalated further by containment in environments unsuited to the needs of the patients and associated risks (1.3.1.).

#### Theme 2: Seeking Alternative Options

In the absence of a diagnosable mental disorder, respondents anticipated that admission on informal basis or through Deprivation of Liberty statutes might take its place. Some worried this would be at the expense of the patient’s rights (2.1.2.).

A strong subtheme emerged around the concern of a growing practice of shunting PwID into Part 3 sections of the MHA and the Criminal Justice System, as criminalising the presenting behaviours for many was felt to be the only way left to access appropriate treatment (2.2.3., 2.2.5.). The potential for this to result in lengthier admissions and ‘warehousing’ of vulnerable individuals in the Criminal Justice System was also acknowledged by respondents (2.2.6.). One respondent expressed an opposing view that a Part 3 sections would at least be preferable to community alternatives in circumstances where a Part 2 section would no longer be an option (2.2.7.). A minority of respondents reflected that responsibility and accountability is important for all people who have capacity to understand the consequences of their actions, whether intellectual disability is present or not, and that the Criminal Justice System may be the most appropriate route in these cases. One participant remarked that in current practice there is a tendency to avoid charging PwID with a criminal offence, in favour of directing them into the hospital system (2.2.1.1.).

#### Theme 3: Introducing Inequities

Concerns emerged around the proposed changes exacerbating the existing challenges for PwID in accessing good quality healthcare. In the absence of a hospital option, respondents hypothesised that the use of psychotropic medication and restrictive practices would increase in the community. Without a suitable service to support the complex needs and presenting risks, PwID could find themselves in a ‘no man’s land’ where none of the available support options would be suitable (3.2.4.). In the absence of S117 aftercare provisions, care packages could become increasingly difficult to source, further reducing the quality of care provided to PwID (3.2.8.).

#### Theme 4: Resources

Respondents identified current gaps in community care provisions as a significant obstacle to successful implementation of the proposed MHA changes. They were clear that community teams are already struggling to meet demand (4.1.4) and would not be able to respond to the increased pressures expected to result from the proposed legislative changes if left in their current state. Investments into staff across all multidisciplinary team (MDT) disciplines were recommended as a priority (4.1.2., 4.1.3.). Alongside this, an increase in service provisions such as forensic community teams and crisis services (4.2.2., 4.2.3.) is needed, in order to offer the right support, contain risks, facilitate smooth discharges from hospital and act as gatekeepers for admission (4.2.4.). Participants also highlighted that closer links between services through collaboration between the intellectual disability services MDT, primary care, and social services are needed to allow professionals to collaborate towards joint, robust, combined health and social care goals (4.3.2.). There was a sense that increased pressures on community intellectual disability teams and social care from the proposed changes would overwhelm the current system, which was identified as already being short-staffed, under-resourced and faced with too many vacancies to cater for the current level of needs (4.1.4.). Participants expressed that a lack of investment has precluded social care provisions from developing in a way which can support the proposed changes, and the challenges in sourcing community placements would be a rate-limiting step in supporting PwID in the community (4.4.7.).

#### Theme 5: Meeting Holistic Care Goals Through the CETR Process

The quality of CETRs was perceived to be unpredictable, with variability in clinical knowledge of panel members creating a misalignment between the patient’s clinical needs and panel recommendations (5.1.3.). Clinicians felt that at times they would have to deviate from the CETR recommendations to provide person-centred care. Preserving clinical autonomy was therefore important, and a potential introduction of a statutory requirement to respond to panel recommendations was described as ‘heavy handed and restrictive’ (5.2.1.). One person highlighted their positive experiences of the CETR process and felt that the proposed changes would leave room for clinical flexibility (5.2.5.). A few participants proposed the introduction of a ‘peer review’ system by other clinicians working in the psychiatry of intellectual disabilities as a way to improve the CETR process. Responders agreed that responsibility for addressing CETR panel recommendations should be shared across the health and social care MDT and service managers, rather than rest solely with the Responsible Clinician. Recommendations can be specific to certain professional groups or entirely within the remit of social care to address, and to place the onus on the responsible clinician in such cases was not felt to be a good use of resources (5.3.1., 5.3.4.).

## Discussion

This study describes the views to the proposed changes to MHA legislation for PwID of psychiatrists working in the field of intellectual disability. The majority of participants (*n* = 53; 64%) reported reasonably good awareness of the proposed MHA reforms, with widespread concern regarding proposed changes, with over half (*n* = 46; 55%) indicating disagreement with the proposed changes, and only 5% (*n* = 4) of participants reporting full agreement. This widely shared concern has been expressed previously in supportive editorials within the profession (Courtenay, 2021; Tromans et al., 2023; Tromans & Biswas, 2021) and has been highlighted to the Parliamentary Joint Committee by leading clinicians (Parliamentlive.tv, 2022).

Subgroup analyses identified an increased awareness of the proposed changes (*p* = .02) and a higher suspicion of unintended consequences of the reforms (*p* = .02) among consultant participants, compared to their non-consultant peers.

Additionally, psychiatrists working partially or entirely in inpatient settings reported increased awareness of the proposed changes (*p* = .007) compared to their colleagues working exclusively in community settings, as well as less agreement with the reforms (*p* = .04), less confidence that 28-day detention was sufficient (*p* = .03), increased belief that behaviours not due to mental health require detention (*p* = .004), less confidence that the reforms provide adequate safeguards (*p* = .004), increased expectations of the reforms having unintended consequences (*p* < .001), and reduced agreement with the MHA changes (*p* = .03). These inpatient clinicians are likely to have more hands-on experience of the challenges associated with the detention of PwID than their peers, due to the nature of their roles. The strength of feeling amongst this group of clinicians in particular should be considered carefully by bodies tasked with appraising the Draft Bill (Draft Mental Health Bill, 2022).

Descriptive thematic analysis of free text survey responses identified five main themes outlining clinicians’ opinions and concerns regarding the proposed changes to the MHA for PwID. Clinicians were concerned that proposed changes may have a detrimental impact on the ability to arrive at appropriate diagnoses and subsequent treatment plans if enacted.

There was extensive concern regarding the potential for mis-diagnosis or overdiagnosis of mental health conditions or mis-use of alternative legal frameworks to secure inpatient treatment, as well as concern regarding the potential for patients being shunted into the Criminal Justice System. These concerns have already been brought to the attention of the Parliamentary Joint Committee in their appraisal of the Draft Bill (Parliamentlive.tv, 2022), and this survey highlights that these significant concerns are shared amongst clinicians across the country.

Concerns raised by clinicians regarding the potential for PwID to be redirected into the Criminal Justice System highlight that this could lead to far lengthier and complicated admissions. The removal of eligibility for Section 117 aftercare may inhibit vital funding streams for appropriate community provisions, effectively lengthening admissions further and reducing the overall quality of care for PwID. In consideration of these concerns, it would be prudent to reflect on the legislative changes enacted in New Zealand in 1992 (Mental Health (Compulsory Assessment and Treatment)Act, 1992), and the subsequent scrutiny of these changes, which highlighted a ‘legislative gap’, reportedly ‘closing the civil entry for PwID’ (McCarthy & Duff, 2019).

Clinicians also reported concerns regarding the increased need for specialised health services and community provisions to safely implement the proposals within the Draft Bill (Draft Mental Health Bill, 2022). Calls for greater investment in robust community provisions for PwID have been repeatedly cited as a priority for government attention for many years (Department of Health, 2001; Mansell, 2007), however many have argued that subsequent investments have not matched the need of PwID. Legislative changes to remove PwID from the scope of the MHA have been proposed as a mechanism to prevent their long-term detention in hospital, however, concerns raised nationally and within our survey data point out that focussing on legislative frameworks as the bottleneck to safe discharges may be ‘missing the boat’, where greater attention should be focussed on investment in community provisions and resources.

Echoing the concerns of experts heard by the Parliamentary Joint Committee in relation to the proposed changes to the CETR process (Parliamentlive.tv, 2022), Clinicians within this survey raised concerns regarding the quality of the CETR process and the potential restrictiveness of the statutory responsibilities. In addition, the currently unaddressed necessity for statutory responsibilities to act on agreements to be placed in the hands of those systems capable of making the changes, rather than putting ownership into the hands of the Responsible Clinician, who may have no power over the acquisition of safe and appropriate community placements.

### Strengths and Limitations

This survey obtained views from psychiatrists working with PwID on an issue of clear relevance to the care of PwID, which has a potential far-reaching impact on their wellbeing and care. To our knowledge, this survey represents the first attempt to collect, analyse, and report the views of psychiatry professionals working in the field of intellectual disability, who have extensive hands-on experience of applying such MHA legislation in their clinical practice.

A conscious decision was made to exclude non-psychiatry clinicians who hold ‘Approved clinician’ responsibilities which allows them to have similar function as psychiatrists with ‘responsible clinician’ competencies. This was to prevent any heterogeneity in the study group. There are very few such non-psychiatrist approved clinicians in England and Wales.

It is possible that those with stronger feelings pertaining to the proposed MHA changes would have been more likely to engage in the survey, though every reasonable effort was made to invite all psychiatrists working with PwID based within England and Wales. The survey return rate suggests that approximately a third of the eligible study cohort responded.

Participants may have felt constrained with regard to the questions posed to them in the survey, and more detailed contextual information pertaining to their views may be obtainable via other methodological approaches, such as focus groups and/or individual semi-structured interviews.

The survey was only available for completion in online format, with no alternative approaches for survey administration available to potential participants, such as paper format or telephone completion. However, psychiatrists working with PwID in England and Wales would have to be digitally literate in order to carry out their clinical roles, so we suspect the impact of digital exclusion would have been fairly minimal among this group.

### Implications for Research, Policy, and Practice

Further contextual information on the views of psychiatrists could be obtained via semi-structured interviews, which would afford participants a greater level of flexibility in discussing areas of greatest import to their own practice, compared to a survey design.

If the proposed MHA changes are put into practice, our survey findings highlight a need to assess the ongoing impact of these changes in relation to PwID, and sufficient flexibility to reverse the changes in the event they cause net harm to this patient group. Thus, there is a clear need for ongoing research in this area. The concerns of the psychiatry profession should clearly be factored into decision making with regard to these proposals, as well as those of other groups, including non-psychiatrist ID professionals, patients, and carers.

## Conclusion

In summary, psychiatrists working with PwID report widespread disagreement with the proposed changes to the MHA with relation to PwID. This disagreement is greater among those working partially or entirely within inpatient settings, compared to their peers working exclusively in the community. These concerns raised by psychiatrists working with PwID across England and Wales echo those raised by experts to the Parliamentary Joint Committee (Parliamentlive.tv, 2022), who have been tasked with appraising the proposals.

## Supplemental Material

sj-docx-1-isp-10.1177_00207640231212112 – Supplemental material for The views of psychiatrists on proposed changes to the England and Wales Mental Health Act 1983 legislation for people with intellectual disability: A national study Supplemental material, sj-docx-1-isp-10.1177_00207640231212112 for The views of psychiatrists on proposed changes to the England and Wales Mental Health Act 1983 legislation for people with intellectual disability: A national study by Samuel Tromans, Gemma Robinson, Alexandra Gabrielsson, Paul Bassett, Indermeet Sawhney, Paraskevi Triantafyllopoulou, Angela Hassiotis and Rohit Shankar in International Journal of Social Psychiatry

sj-docx-2-isp-10.1177_00207640231212112 – Supplemental material for The views of psychiatrists on proposed changes to the England and Wales Mental Health Act 1983 legislation for people with intellectual disability: A national study Supplemental material, sj-docx-2-isp-10.1177_00207640231212112 for The views of psychiatrists on proposed changes to the England and Wales Mental Health Act 1983 legislation for people with intellectual disability: A national study by Samuel Tromans, Gemma Robinson, Alexandra Gabrielsson, Paul Bassett, Indermeet Sawhney, Paraskevi Triantafyllopoulou, Angela Hassiotis and Rohit Shankar in International Journal of Social Psychiatry

sj-docx-3-isp-10.1177_00207640231212112 – Supplemental material for The views of psychiatrists on proposed changes to the England and Wales Mental Health Act 1983 legislation for people with intellectual disability: A national study Supplemental material, sj-docx-3-isp-10.1177_00207640231212112 for The views of psychiatrists on proposed changes to the England and Wales Mental Health Act 1983 legislation for people with intellectual disability: A national study by Samuel Tromans, Gemma Robinson, Alexandra Gabrielsson, Paul Bassett, Indermeet Sawhney, Paraskevi Triantafyllopoulou, Angela Hassiotis and Rohit Shankar in International Journal of Social Psychiatry

sj-docx-4-isp-10.1177_00207640231212112 – Supplemental material for The views of psychiatrists on proposed changes to the England and Wales Mental Health Act 1983 legislation for people with intellectual disability: A national study Supplemental material, sj-docx-4-isp-10.1177_00207640231212112 for The views of psychiatrists on proposed changes to the England and Wales Mental Health Act 1983 legislation for people with intellectual disability: A national study by Samuel Tromans, Gemma Robinson, Alexandra Gabrielsson, Paul Bassett, Indermeet Sawhney, Paraskevi Triantafyllopoulou, Angela Hassiotis and Rohit Shankar in International Journal of Social Psychiatry
