# Supplementary material for: The views of psychiatrists on proposed changes to the England and Wales Mental Health Act 1983 legislation for people with intellectual disability: A national study
Source: Int J Soc Psychiatry. 2023 Nov 24;70(2):388–401. doi: 10.1177/00207640231212112 (PMC10913297; doi:10.1177/00207640231212112)
Supplement: sj-docx-2-isp-10.1177_00207640231212112 – Supplemental material for The views of psychiatrists on proposed changes to the England and Wales Mental Health Act 1983 legislation for people with intellectual disability: A national study [file sj-docx-2-isp-10.1177_00207640231212112.docx]

Mental Health Act survey

**Views of psychiatrists working with people with intellectual disabilities (PwID) on proposed MHA reforms**

**Dear colleague**

Recently the Joint Committee on the draft mental health bill (https://committees.parliament.uk/work/6888/draft-mental-health-bill/publications/) has made recommendations on changing the criteria for detention under section 3 for people with intellectual disabilities (PwID). This follows the publication of the White paper on this matter. These recommendations can have a significant impact on the working practices of psychiatrists who work with PwID.

Under the proposals, detention using the criteria of abnormally aggressive or seriously irresponsible conduct will not be considered warranting compulsory treatment under section 3 of the Mental Health Act (MHA). This is in the absence of a co-occurring mental illness.

This is a brief survey of psychiatrists who work primarily with adults with PwID to help understand what the practice, challenges and possible barriers will be if these the section 3 changes go ahead. It would allow us to gain insight to the unique experiences and challenges by psychiatrists working in the field and canvass support to respond suitably on behalf of the patients, families and carers we care for and the services we work for.

The collated results and analysis of the survey will be fed back via suitable channels (example RCPsych ID faculty blog etc.)

All questions are optional and the majority are multiple choice. We envisage the survey to take a maximum of 8 to 10 minutes to complete. The survey is designed to keep your replies anonymous. We would presume informed consent if you submit the survey.

We appreciate this is a busy time for you and you could be getting various other requests for information. We do hope this survey output can be representative of your needs and views and make a direct impact on service delivery. We are very grateful for your consideration of this.

# What is your role/specialism?

## *Mark only one oval.*


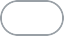
 Consultant psychiatrist working with PwID (adults)
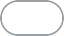
 ST4-6 (Higher) trainees working with PwID


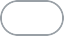
 Speciality and Associate Specialist (SAS) doctors working with PwID
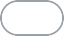
 others

# How many years of experience do you have working with PwID (adults)

## *Mark only one oval.*


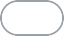
 less than 5 years
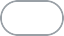
 6-10 years


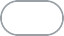
 11-15 years


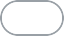
 More than 15 years

# What settings do you currently work in?

## *Mark only one oval.*


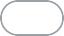
 Hospital (inpatients)


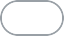
 community (outpatient) based
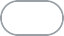
 both hospital and community

# What % of your working week is work with PwID?

## *Mark only one oval.*


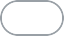
 less than or equal to 25%
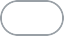
 26-50%


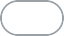
 over 50%

# In a typical year how many PwID do you see who are detained on section 3 of the MHA for abnormally aggressive or seriously irresponsible conduct (without a co-occurring major mental illness).

## *Mark only one oval.*


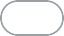
 none
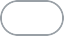
 1-3


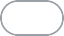
 4-6


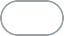
 7-10


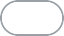
 11-20


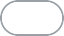
 more than 20

# Prior to this survey how aware were you of the reforms to the MHA for PwID?

*Mark only one oval.*

Not aware at all

1

2

3

4

5

Fully aware

# Indicate your overall level of agreement to the MHA reform proposals for PwID specific to the change of withdrawal of using the criteria of abnormally aggressive or seriously irresponsible conduct for warranting compulsory treatment under section 3 of the Mental Health Act (MHA)

*Mark only one oval.*

Fully disagree with the proposed change

1

2

3

4

5

Fully agree with the proposed change

# How confident are you that the 28 days duration for detention allocated in Section 2 of the MHA is enough time to ascertain if there is an underlying mental health condition driving the concerning behaviour/s?

*Mark only one oval.*

Not confident

1

2

3

4

5

Fully confident

# How strongly do you believe behaviours in PwID which are not due to an underlying mental health disorder should require detention under section 3 of the MHA if they are a risk to themselves or others?

*Mark only one oval.*

Not at all

1

2

3

4

5

Very strongly

# How confident are you of the proposed reforms providing adequate safeguards for PwID when they do not have a co-occurring mental health condition?

*Mark only one oval.*

Not confident at all

1

2

3

4

5

Fully confident

# Do you expect any unintended consequences (negative or positive) of the proposals?

## *Mark only one oval.*


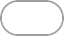
 Yes
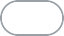
 No
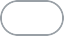
 Maybe

# please describe the reasons for your choice above.

1. The proposal to change the MHA for PwID is under Part II (i.e., ‘civil’ sections) and not Part III (i.e., sections through the criminal justice system). Is this agreeable?

## *Mark only one oval.*


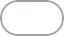
 yes
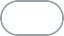
 No


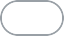
 Not sure/maybe

# Please provide reasons for our choice above

1. Could there be unintended consequences on the Criminal Justice System as a result of the proposals to reform the way the MHA for PwID?

## *Mark only one oval.*


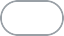
 There can be positive consequences for PwID
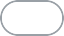
 There can be negative consequences for PwID


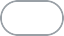
 There can be no significant consequences for PwID


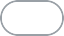
 There can be both positive and negative consequences for PwID
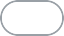
 It is difficult to envisage the consequences

1. Please give reasons for your response above
2. The Commission recommend significant resourcing before any changes to the MHA for PwID are implemented. What resources in inpatient and community would you require in your local situation to be confident of delivering the MHA changes? Please describe upto 5
3. The White Paper proposes a statutory requirement that the Responsible Clinician considers the findings and recommendations of Care, Education and Treatment Reviews (CETRs) in the patient's care and treatment plan. Deviations from the recommendations should be justified and explained by the RC. What are your views on this?

*Mark only one oval.*

Fully Disagree

1

2

3

4

5

Fully Agree

# Please could you provide reasons for your position taken above

This content is neither created nor endorsed by Google.

[
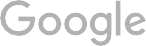
](https://www.google.com/forms/about/?utm_source=product&utm_medium=forms_logo&utm_campaign=forms) [Forms](https://www.google.com/forms/about/?utm_source=product&utm_medium=forms_logo&utm_campaign=forms)
