# Supplementary material for: The views of psychiatrists on proposed changes to the England and Wales Mental Health Act 1983 legislation for people with intellectual disability: A national study
Source: Int J Soc Psychiatry. 2023 Nov 24;70(2):388–401. doi: 10.1177/00207640231212112 (PMC10913297; doi:10.1177/00207640231212112)
Supplement: sj-docx-4-isp-10.1177_00207640231212112 – Supplemental material for The views of psychiatrists on proposed changes to the England and Wales Mental Health Act 1983 legislation for people with intellectual disability: A national study [file sj-docx-4-isp-10.1177_00207640231212112.docx]

**Supplementary Table 1.** Thematic analysis data table with all participant comments. Please note that the quoted text is copied verbatim from the survey, with no grammatical/spelling errors corrected, though in some instances the full response has not been presented owing to its length.

| Themes | Participant comments |
| --- | --- |
| 1. Impact on diagnosis and treatment | |
| - 1. Distortion of the diagnostic process | - 1.1.1.“Section 12 approved doctors may feel inclined to exaggerate traits to the extent that a secondary mental health disorder can be proposed in order to keep the person safe in hospital”. - 1.1.2. “Many PwID will pick up unnecessary labels with overdiagnosis/misdiagnosis of major mental illnesses”. - 1.1.3. “There will be an increase in diagnosis of mental illness in PwID to justify ongoing detention”. |
| - 1. Understanding complex presentations – need for time | - 1.2.1.“I have seen a number of cases where it has taken significantly longer than 28 days to get an understand of the causes of the aggression, understand their needs and settle them sufficiently (sometimes this has been in longer term segregation)”. - 1.2.2. “Some time you need to observe the patient , in hospital to determine if the presention is behavioral or mental illness , so time under the MHA will allow us to assess and treat”. - 1.2.3.“In our forensic unit we use minimal medication but have come across patients whereby their behaviour has significantly changed for the better after period of stabilisation on medication and it is clear in retrospect they had a mental illness but required at least 6 months to settle and the picture to become clear”. |
| 1.3. Containing risk | - 1.3.1.“As a result of inability to get the patient detained, placements may seek to involve police instead which could result in placement in custody. As a result of being held in an inappropriate environment there could be significant risks”. - 1.3.2.“Increase pressure on A&E, GP's, police, community teams and social care from these complex patients who could pose immediate harm to themselves / the public / family / staff which could be severe or even life altering if they are not in a suitable place with specialist staffing training in MHA” - 1.3.3.“PwID who present with abnormally aggressive or seriously irresponsible conduct (without a co-occurring major mental illness) would continue to pose significant risks to themselves and others if they are not provided with the means and appropriate legal safeguards to enable adequate time longitudinal assessment and treatment where needed before safe transition back into the community. Detention under Section 2 of the MHA is unlikely to provide this”. |
| 2.Seeking alternative options | |
| 2.1. Lack of safeguards | - 2.1.1. “Patients may end up being detained under different legislations which does not have same safeguards”. - 2.1.2. “May result in unlawful detention in hospitals without an appropriate legal framework and possible overuse of DOLS”. - 2.1.3.“A negative chaotic and rushed decision to treat them informally or under DoLS as there are no resources in the community to manage such situations”. - 2.1.4. “If the MCA starts to be used more to enable PwID to access hospital treatment without the MHA option, then PwID will have fewer rights as they have no right to a Tribunal or other MHA safeguards to their admission”. |
| 2.2. Criminalisation of behaviour | - 2.2.1.“More vulnerable people end up being treated under criminal justice system rather than mental health services”. - 2.2.2.“Patients being prosecuted so that they can have a criminal section”. - 2.2.3. “Means people have to offend before they get appropriate treatment in hospital”. - 2.2.4.“No internal logic; paradoxical diversion of vulnerable people to the prison system”. - 2.2.5.“Well, there is really a thin line between challenging and offending behaviour and I think eventually PwID who have challenging behaviours will negotiate forensic routes to access needed treatments”. - 2.2.6. “More people may be shunted into Part 3 of the act, due to Part 2 becoming unavailable to them. These are far more restrictive powers and will make the so called “warehousing of PwID” a far bigger and lengthy problem”. - 2.2.7. “I think all PwID should have access to inpatient treatment when needed, under Part 2 and Part 3, for the reasons above. At least if we keep Part 3, then some PwID will be able to still access this treatment”! |
| 2.2.1. Accountability | - 2.2.1.1 “It might be entirely appropriate for PWID to get charged for their behaviour and this often doesn’t happen as they will end up in the hospital system”. - 2.2.1.2. “If someone is capacitous for their actions they should face the CJS (not MHA Part III) regardless of whether they have an LD or not” |
| 3.Introducing inequities | |
| 3.1. Discrimination and stigma | - 3.1.1.“I think this is negative discrimination as patients involved in criminal justice have inequitable access to services to patients who haven't a forensic history”. - 3.1.2.“It is discriminatory and will stigmatise people in to two categories of 'offender' and 'non-offender'. Services will not become involved with a person until they have committed and offence leading to later interventions”. |
| 3.2. Impact on existing health inequalities | - 3.2.1.“There is a possibility PwID will be subjected to more restrictive practices”. - 3.2.2.“People with LD will get an inferior service compared to general population”. - 3.2.3. “PwID should have a right to the same access to high quality inpatient care as those without ID (dementia for example) - this proposed legislation takes away this treatment option for PwID”. - 3.2.4. “People with ID ending up in "no man's land" - too risky for community, ineligible for hospital, not detained by police - this leaves PwID, their families, carers, and professionals all vulnerable to additional stress and claims of neglect”. - 3.2.5. “The reason for the changes is to improve equity and autonomy for PwID. To allow detention under part 3 and disallow it under Part 2 is a blatant error in logic if this was what was intended”. - 3.2.6. “Undue pressure on community consultants to prescribe psychotropic medication and many for whom appropriate treatments (psychosocial, environmental, etc) is available in hospital won't be able to access it”. - 3.2.7.“Excessive use of chemical and physical restraint in community settings”. - 3.2.8. “Harder to source discharge care package (more so if S117 funding is not available)” |
| 3.3. Equality and rights | - 3.3.1.“I think it is right that adults with learning disability should have the same rights and responsibility as other members of society so some of this reform is in the right direction if paired with appropriate support within LA, police and judicial system”. |
| 4. Resources | |
| 4.1. Staffing availability and expertise | - 4.1.1.“More learning disability nurses”. - 4.1.2. “Full time OT, SALT, psychology and people trained in sensory assessments and PBS plans in every community and inpatient team”. - 4.1.3.“Community services requires more specialist staff to provide a comprehensive mental health service to prevent PwID commiting offences” - 4.1.4. “More people - staff in services. We cannot recruit to vacant posts locally for professional staff. Services need to concentrate on recruiting to current levels of provision before even contemplating expansion of services”. |
| 4.2. Community service provisions | - 4.2.1. “This will mean more and more community forensic teams will need to be established which can take up patients with what could be not a threshold criteria for patients usually seen in forensic teams. Many of our patients with offending behaviours are classified as challenging behaviours and the police and CPS don't take action and leave their management to the local mental health/learning disability services” - 4.2.2. “Intensive community LD support teams / crisis services which don't exist locally at present” - 4.2.3. “More community forensic teams will need to be established which can take up patients with what could be not a threshold criteria for patients normally seen in forensic services”. - 4.2.4.“The main requirement is significant investment in community provisions that enable patients to be kept safely in the community. This is the common barrier to discharge and often the reason for admission in the first place”. - 4.2.5.“A possible positive might be de-medicalising presentations and reducing hospital length of stay but this will only happen if adequate community placements are available with capacity to move people on from hospital (or prevent hospital admission)”. - 4.2.6. “There would possibly need to be respite beds aimed at adults with very challenging behaviours with access to specialised professionals who can help with further assessment”. - 4.2.7.“Unless appropriate and robust community provisions are made a priority, the proposed bill changes will result in mid-diagnosis, mis-direction and mis-use of alternative legal frameworks such as the MCA”. |
| 4.3. Systems perspective | - 4.3.1.“Better links with forensic teams and ability to consult with them”. - 4.3.2.“Collaborate health and social care commissioners to build well-resourced 'step down/up' placements in community with 24x7 experienced staffing and MDT availability through the week”. - 4.3.3.“Sharing of notes between different trusts (a lot of OOA admissions in ID)”. - 4.3.4.“GP input during assessment period for each patient and continues during admission”. |
| 4.4. The role of social care provisions | - 4.4.1.“The proposal assumes that all other agencies involved are working as intended which is not the case - there are a shortage of skilled providers, a shortage of alternatives to hospital community placements and limited housing options for PwID”. - 4.4.2.“I have not seen any investment in social care to ensure it develops in line with the proposed MHA changes”. - 4.4.3.“The biggest resource required is improved availability of skilled community placements where PwID and behaviours that challenge can be safely managed”. - 4.4.4. “Robust social care provisions which at present are practically nonexistent” - 4.4.5. “The proposals are not workable in the current and future climates and people with ID will suffer needlessly. I hope the proposers are prepared to accept the consequences of their misguided proposals and actions”. - 4.4.6. “Develop a list of competences/skills expected in specialist community placements which are enforceable by local authorities. End practice of '28-day notice' given by community providers, which is followed by getting the PwID to A&E and a resultant hospital admission” - 4.4.7.“The current community resources make it IMPOSSIBLE to find community alternatives within 28 days, unless drastic changes are made to the way local authorities respond by allocating social workers with knowledge of ID; approach providers; agree funding; and more widely, stimulate community provider market”. |
| 5. **Meeting holistic care goals through the CETR process** | |
| 5.1. Disconnect between panel members and clinicians | - 5.1.1.“The quality of CCTR's and how they are chaired is unpredictable. Other people in CCTR may not have enough knowledge of psychopathology in ID to make formulation and treatment plan”. - 5.1.2. Make CTR's more relevant by drawing on active clinicians working in ID services - consider 'peer review' by clinicians working in other ID hospitals. - 5.1.3.“These recommendations are made by usually those who are not directly involved in patient's care and have only heard what is being said in a CTER/CTR. The practical situation could be much different in terms of the nature of a patient's presentation, challenging behaviours, expectations from clinicians, and the availability of resources”. - 5.1.4.“Extremely variable quality of panel members; fundamental unfairness in inability to challenge panel decisions; no appeals process”. - 5.1.5.“Well I think the RC is ultimately responsible for patient care and often may need to do things different from CeTr recommendations to ensure patient centred care is provided”. |
| 5.2. Imposing recommendations | - 5.2.1.“I would hope all RCs consider a person's Care and treatment plans already. I don't think making it statutory would change this and feels a bit heavy handed to make it a requirement and will no doubt mean an extra form to sign. I don't feel this would make any difference in practice to patient care”. - 5.2.2.“Clinical autonomy is key for good psychiatric practice and CETR is far too prescriptive” - 5.2.3.“Punitive or statutory requirements do not lead to good practice, but lead to unintended consequences”. - 5.2.4.“It should be the other way around that the clinical team's recommendations need to be met by commissioners and decision makers”. - 5.2.5.“I find CTRs excellent, and that the panel is in general knowledgeable and helpful. It is important that their recommendations should be followed. The proposed change still allows for an RC to deviate - for example, sometimes the panel does come up with a rogue suggestion but this is covered in the proposed deviation-clause. At the moment, the following or not of CTR recommendations is too dependent on the particular approach of the RC”. |
| 5.3. Shared responsibility and accountability | - 5.3.1. “This should be shared responsibility across the MDT, there are recommendations which are specific to say SLT or OT and it isn’t a good use of RC time to be chasing up other MDT members on their actions”. - 5.3.2. “Responsible Clinician should be responsible for only treatment , care aspect & education is responsibility of the Social Services and above all, the Trust/ Hospital looking after the patient”. - 5.3.3. “It (CETR recommendations) should be everyone's responsibility and not just the RC. If the responsibility is to be held by one person, this should be the team or clinical manager who has more authority over an MDT than the RC. Either that or empower RC's and enable them to hold the MDT to account. - 5.3.4. “The RC should explain why deviations from care plans are made, but the government need to accept that in many cases, the reason why the patient cannot be discharged is due to lack of suitable community provisions. The RC can and should only be held responsible for those elements of the care plan that he or she is empowered to be able to deal with. This would not include the sourcing and funding of community provisions”. |
